# “Where-There-Is-No-Psychiatrist Integrated Personal Therapy” among Community-Dwelling Older Adults: A Randomized Pilot Study

**DOI:** 10.3390/ijerph18189514

**Published:** 2021-09-09

**Authors:** Shefaly Shorey, Ee Heok Kua, Wilson Tam, Valerie Chan, Yong Shian Goh, Hong Meng Lim, Lina Hsiu Kim Lim, Cheong Sing Tian, Rathi Mahendran

**Affiliations:** 1 Alice Lee Centre for Nursing Studies, Yong Loo Lin School of Medicine, National University of Singapore, Singapore 117597, Singapore; nurtwsw@nus.edu.sg (W.T.); val_chan@live.com (V.C.); nurgys@nus.edu.sg (Y.S.G.); 2 Department of Psychological Medicine, Yong Loo Lin School of Medicine, National University of Singapore, Singapore 117597, Singapore; pcmkeh@nus.edu.sg (E.H.K.); cheong_sing_tian@nuhs.edu.sg (C.S.T.); pcmrathi@nus.edu.sg (R.M.); 3 Department of Physiology, National University of Singapore, Singapore 117593, Singapore; phslimh@nus.edu.sg (H.M.L.); phslhkl@nus.edu.sg (L.H.K.L.)

**Keywords:** solution-focused brief therapy, older adults, mental health, mindfulness

## Abstract

In Singapore, many older adults suffer from subsyndromal depression and/or subsyndromal anxiety, which can negatively impact their physical and mental well-being if left untreated. Due to the general public’s reluctance to seek psychological help and the low psychiatrist-to-population ratio in Singapore, this study aims to examine the preliminary efficacy, perceptions, and acceptability of a trained volunteer-led community-based intervention on community-dwelling older adults. Twenty-one participants (control: *n* = 11; intervention: *n* = 10) completed the randomized pilot study. A mixed-methods approach (questionnaires, semistructured interviews, examining blood samples, intervention fidelity) was adopted. No significant differences were found between the intervention and the control groups in depression, anxiety, life satisfaction, friendship, and quality of life. However, there was a positive change in quality-of-life scores from baseline to 6 months in the intervention group. The control group had significantly higher cortisol levels and lower annexin-A1 levels at 6 months, while the intervention group did not. Three themes emerged from the interviews: (1) impact of the intervention on older adults’ well-being, (2) attitudes toward intervention, and (3) a way forward. However, intervention efficacy could not be established due to small sample size caused by the coronavirus pandemic. Future randomized controlled trials should evaluate volunteer-led, technology-based psychosocial interventions to support these older adults.

## 1. Introduction

The global population is aging, and some of the rapidly aging countries are in Southeast Asia [1]. In Singapore, the proportion of older adults almost doubled from 8.8% in 2009 to 14.4% in 2019 [2]. As older adults face special physical and mental health challenges, it is imperative to have impactful paradigm shifts in healthcare delivery for health promotion and protection for this vulnerable population. According to the World Health Organization [3], over 20% of adults aged 60 and above suffer from psychological or neurological disorders, with approximately 7% and 3.8% of them being affected by depressive and anxiety disorders, respectively.

In Singapore, many older adults have depressive and/or anxiety symptoms that fail to fulfill the diagnostic criteria for clinical disorders [4,5,6], otherwise known as subsyndromal depression (SSD) and/or subsyndromal anxiety (SSA). The prevalence rate of older adults with SSD in Singapore has risen from 9.6% in 2008 to 13.4% in 2016 [6], while the prevalence rate of SSA is unclear. A previous review on community samples has reported prevalence rates of SSA ranging from 15% to 52.3% [5]. Both SSD and SSA could coexist, affect one’s quality of life, and exacerbate other health conditions, putting one at higher risk of cardiovascular disease and death [7,8], declining cognitive function [9], and suicide [10]. Therefore, it is essential to detect and develop novel programs to treat older adults with SSD and/or SSA to prevent the worsening of symptoms and subsequent consequences. 

Apart from depression and anxiety, high stress levels can lead to premature aging, increasing risks for cardiovascular diseases [11,12]. Stress enhances stress hormones, including cortisol, a corticosteroid hormone produced by the adrenal glands, which is important in the maintenance of blood pressure, fluid balance, and inflammation [13]. Optimal amounts of cortisol can be lifesaving, while chronic release of cortisol can lead to immunosuppression as the body becomes resistant to the actions of cortisol and inflammation ensues [13]. Annexin-A1, an anti-inflammatory protein that has inflammation pro-resolving properties [14], may be reduced during stress. Given the association between stress and physical health, it is essential to examine stress biomarkers when studying mental health among older adults.

In Singapore, mental health is often regarded as a taboo topic [15,16]. Adults with mental health conditions often delay seeking help, and mental health services are not fully utilized by the population [15]. Moreover, the lack of awareness and unfamiliarity with available resources and interventions led to the rejection of standard psychiatric and/or psychological treatments [17]. Furthermore, with the low psychiatrist-to-population ratio of only 2.6 per 100,000 compared to other developed countries with a higher ratio such as Australia (14 per 100,000) and the United Kingdom (11 per 100,000) [18], it is difficult to reach out to all older adults in need [18,19]. Hence, it is necessary to mediate cultural barriers and population preferences to develop mental health management strategies that are acceptable and accessible. 

Community-based programs can provide more accessible mental health care for older adults [20]. Community-based mental health programs have been associated with improved depressive and overall psychiatric symptoms in older adults [20]. Moreover, a recent community-based psychosocial intervention program, consisting of tai chi exercise, art therapy, mindfulness training, and music reminiscence therapy, has shown effectiveness in reducing SSD and SSA symptoms among older adults [19]. As community-based mental health programs have shown promising effectiveness in alleviating mental health conditions, they should be explored further. 

In this randomized pilot study, a trained lay volunteer-led community-based intervention, “**W**here-there-is-no-psychiatrist **I**ntegrated **P**ersonal **T**herapy” (WIPT), integrated solution-focused brief therapy (SFBT) involving psychoeducation and structured life review therapy to assist community-dwelling older adults and evaluated mindfulness-based training developed by experts. SFBT focuses on strengths and solutions rather than problems and deficits and provides a framework for doing brief therapy in a managed environment [21]. A recent review reported that SFBT was capable of treating depression especially among older adults [21]. On the other hand, mindfulness, which originated from Buddhist practices, defined as the process of attending to the present moment’s experience without judgment [22], has been widely integrated into psychological therapies and treatments. Recent studies have reported the effectiveness of mindfulness in reducing depression, anxiety, and worry among adults and older adults [23,24]. While SFBT prepares the older adults for achieving future goals, mindfulness helps them to remain in the present while enhancing their emotional well-being by developing beneficial therapeutic qualities such as acceptance, attention, compassion, equanimity, and presence that enrich and enliven the older adults to avoid further stress. Therefore, given current evidence on the effectiveness of SBFT and mindfulness in improving mental health outcomes, this research aimed to combine both strategies and examine the efficacy of a multimodal and novel intervention design in improving mental health among older adults.

Overall, this study aims to (i) examine the preliminary efficacy of the WIT intervention in reducing SSD and/or SSA symptoms among community-dwelling older adults (primary outcome); (ii) examine the efficacy of WIT in improving life satisfaction, social connectedness (friendship), quality of life, and stress- and anxiety-related inflammatory outcomes (cortisol, annexin-A1, and interleukin-1β) among older adults with SSD and/or SSA (secondary outcomes); and (iii) evaluate the older adults’ perceptions and acceptability of the intervention through semistructured interviews.

## 2. Methods

### 2.1. Study Design

This study was conducted from September 2019 to March 2020 at a senior activity center located in Singapore. A parallel group RCT experimental design was used. The study was approved by the National University of Singapore Institutional Review Board (NUS-IRB Reference Code: LH-19-029) and registered on ClinicalTrials.gov (NCT04927026). 

### 2.2. Participant Recruitment

According to Browne’s rule of thumb [25] on the use of a pilot sample for sample size determination, a minimum sample of 60 older adults (at least 30 older adults per group) was needed in this study. Participants were recruited from a larger Ageing in a Community Environment Study (ACES) cohort [26,27] with the following inclusion criteria: (i) community-dwelling older adults, (ii) aged between 60–95 years old, (iii) understood and spoke English and/or Mandarin, (iv) Mini-Mental State Examination score ≥ 24 as assessed by a trained nurse, (v) showed symptoms of SSD and/or SSA, and (vi) were able to attend at least 80% (five out of seven sessions) of the intervention sessions. The Geriatric Depression Scale (GDS) and the Geriatric Anxiety Scale (GAI) were used to determine whether the older adults showed symptoms of SSD (GDS score between one and five) and/or SSA (GAI score between three and 10). Since the main study recruited older adults who lived near a senior activity center, a study site located on the west side of Singapore, the same study site was used for this study.

In September 2019, a research assistant (RA) contacted eligible older adults via phone calls and/or WhatsApp messages. The older adults were briefed on the study details and given ample time to ask questions. Those who expressed interest in the study were asked to attend a face-to-face meeting at the center in order to obtain written informed consent and distribute participant information sheets. They were then randomly assigned into either the intervention or control group. The Research Randomizer was used to randomly generate two sets of numbers to differentiate the two groups. The numbers were prepared by the RA and placed into an opaque envelope. The number selected by each older adult which corresponded with the generated set of numbers determined the group they were assigned to. The study consisted of three rounds; every round consisted of about 20 subjects, and there would be a maximum of 10 participants in the intervention group. At the end of September 2019, a total of 21 older adults were recruited, and they formed the first batch to kick-start the WIPT intervention in October 2019. At the end of January 2020, another seven older adults (second batch) were recruited. However, due to the coronavirus disease 2019 (COVID-19) pandemic, the second batch of older adults were unable to undergo the intervention due to the closure of the senior activity center, and hence repeated post-tests (follow-up at 3 and 6 months post-recruitment) and participant recruitment ceased. Therefore, only the first batch of older adults (*n* = 21) completed the baseline and repeated post-tests, and the study was stopped in March 2020 due to the imposed COVID-19 lockdown. 

### 2.3. Procedures and Intervention

This study was divided into two phases. Phase I involved the development of the multimodal WIPT intervention, and Phase II involved the delivery and evaluation of the WIPT intervention. The details of Phase I are available in Table S1.

The WIPT intervention, comprising face-to-face mindfulness training and SFBT, was delivered through group activities at a senior activity center located in the west region of Singapore. In any case where participants were confused or had queries, one-on-one guidance was provided. A bilingual female lay volunteer was trained by the principal investigator to deliver the SFBT via interactive sessions. The same volunteer delivered all the seven intervention sessions. A mindfulness practitioner delivered the mindfulness training sessions. The intervention was delivered in both English and Chinese to cater to the needs of the participants, and lay language was used for ease of understanding. Hardcopy training handouts were given to the older adults after each session.

Baseline questionnaires and blood samples were collected together with informed consent when interested participants attended the face-to-face briefing session at the senior activity center. A phlebotomist was engaged to assist in the blood-drawing, and infection control measures (e.g., use of biohazard bags for disposal) were put in place too. Blood sampling took place at noon (12 p.m.) in a meeting room at the senior activity center at baseline and 6 months post-recruitment. At 3 and 6 months post-recruitment, the RA collected follow-up data via hardcopy self-reported questionnaires from the older adults at the senior activity center. At 3 months post-recruitment, all the older adults in the intervention group were contacted, and their interest in participating in a face-to-face interview to explore the acceptability of the intervention was sought. The individual semistructured interviews were scheduled and conducted by the RA in either English or Mandarin using a semistructured interview guide. Each interview lasted for approximately 30 min and was audio-recorded. At 6-month post-recruitment, follow-up data via self-administered questionnaires and blood samples to measure the stress- and anxiety-related inflammatory outcomes were collected. 

The older adults in the control group were supposed to receive the intervention from the same trainers after the 6-month post-recruitment follow-up. However, due to the COVID-19 pandemic, the project ceased and the control group did not receive the intervention. Hence, the control group only completed the baseline and 3- and 6-month post-recruitment follow-up. 

### 2.4. Outcome Measures

The demographic data of older adults were collected at baseline, upon submission of the informed consent forms using self-reported questionnaires. The primary outcomes (symptoms of depression and anxiety) and secondary outcomes (life satisfaction, social connectedness in terms of friendship, and quality of life) of the older adults were measured at baseline and at 3-month (post-test I) and 6-month post-recruitment follow-up (post-test II) using a face-to-face self-reported questionnaire as follows:

#### 2.4.1. Geriatric Depression Scale (GDS) 

The original 15-item version of the GDS included a dichotomous format for participants to select either “Yes” or “No” in response to 15 statements [28]. Five statements were negatively phrased and the scores for those five were reversed for analysis. The maximum score that could be obtained was 15 points; a high score on the scale indicates severe depressive symptoms. Based on previous research, a cut-off score of 5 and below was used to detect SSD [19,29]. The GDS demonstrated satisfactory internal consistency across multiple studies, with Cronbach’s α ranging from 0.80 to 0.92 [30,31].

#### 2.4.2. Geriatric Anxiety Inventory (GAI)

The 20-item GAI adopted a dichotomous format for participants to select either “Agree” or “Disagree” [32] to assess the anxiety symptoms in older adults. The maximum score that could be obtained was 20 points; a high score indicates more anxiety symptoms. A score range of 3 to 10 was used to determine presence of subsyndromal anxiety [19]. The GAI demonstrated satisfactory internal consistency of Cronbach’s α = 0.91 [32]. 

#### 2.4.3. Satisfaction with Life Scale (SWLS)

The 5-item SWLS [33] that included a 7-point Likert scale (1 = “Strongly Disagree”; 7 = “Strongly Agree”) was used to assess the older adults’ satisfaction with life as a whole. The maximum score that could be obtained was 35 points; a high score indicates a high level of satisfaction with life. The SWLS demonstrated satisfactory internal consistency of Cronbach’s α ranging from 0.79 to 0.89 [33,34,35]. 

#### 2.4.4. Friendship Scale (FS)

The 6-item FS [36] was used to assess the level of social connectedness or social isolation among the older adults. The older adults had to select an answer out of the five options (1 = “Not at All”; 5 = “Almost Always”). Of the six items, three were negatively phrased and the scores indicated on the participants’ statements were reversed. The maximum score that could be obtained was 30 points; a high score indicates a high level of social connectedness. The FS showed satisfactory internal consistency of Cronbach’s α = 0.83 [36]. 

#### 2.4.5. World Health Organization Quality of Life OLD (WHOQOL-OLD BREF) 

The 13-item WHOQOL-OLD [37] was used to assess the quality of life in older adults. The older adults had to select an answer based on a 5-point rating scale (1 = “Not at All”; 5 = “Very Satisfied”). The maximum score was 65 points, and a high score indicates a high quality of life. The WHOQOL-OLD BREF showed a satisfactory internal consistency of Cronbach’s α ranging from 0.77 to 0.91 [37,38].

#### 2.4.6. Blood Stress Biomarkers 

Blood samples were collected (at the same times at noon) in lithium heparin tubes at baseline and 6 months post-recruitment. The tubes were transported to the lab at the National University of Singapore. Plasma was collected after spinning the tubes at 1200 rpm and stored at −20 °C for further analysis. The between- and within-group analyses were conducted using the enzyme-linked immunoassay test in which three markers from the blood samples, namely cortisol, annexin-A1, and interleukin-1-β, were examined according to the manufacturer’s instructions by LL.

### 2.5. Data Collection and Analyses

IBM SPSS Statistics for Windows v27.0 (IBM Corp., Armonk, NY, USA) was used to analyze the data. Descriptive statistics were used to summarize the participant demographics and outcome variables using mean and standard deviations for continuous variables and percentages for categorical variables. ANOVA was used to compare baseline continuous variables between groups, and chi-square was used to compare baseline categorical variables. Due to the small sample size, between-group (intervention versus control) differences in the changes in outcome scores from baseline to 3 months and baseline to 6 months were analyzed using Mann–Whitney *U* test, and within-group (control and intervention groups) differences were analyzed using Wilcoxon signed-rank test to show changes in outcomes scores from baseline to the 3- and 6-month follow-up. Statistical significance for all analyses was set at *p* < 0.05. 

The audio recordings and nonverbatim data of the interviews were transcribed and then analyzed using thematic analysis [39]. The transcribed data were first classified into different categories before being collated to form subthemes, where they were reviewed and combined to form themes. These themes described the older adults’ opinions on the strengths and weaknesses of the intervention and ways to improve it. Data analysis was conducted by two independent reviewers, and any discrepancies were resolved using discussions. 

## 3. Results

### 3.1. Recruitment and Participants’ Characteristics

**Figure 1:**
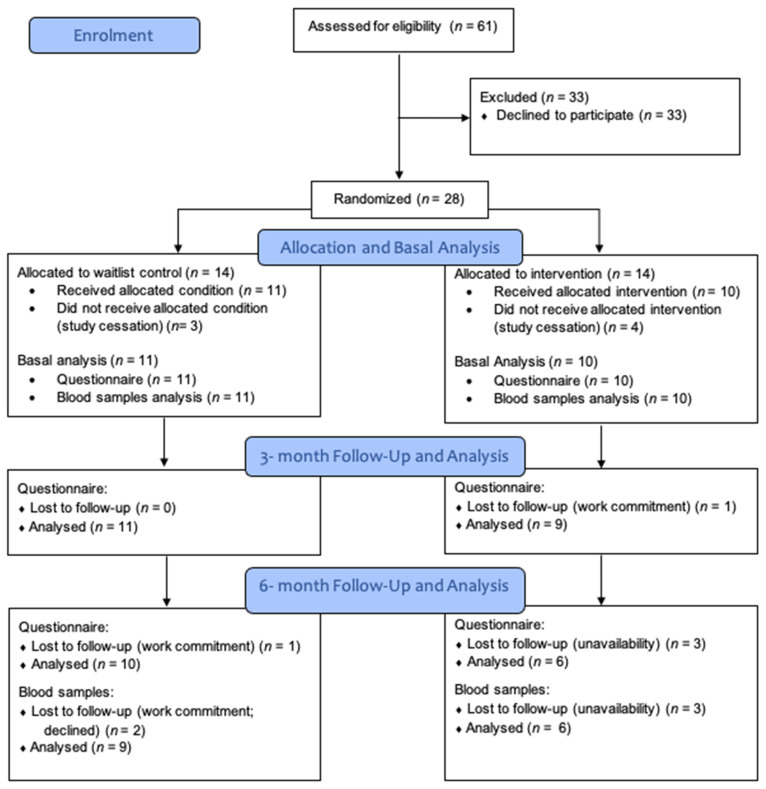
Participant flow chart.

A total of 61 older adults who fulfilled the eligibility criteria were contacted. Thirty-three of them declined to participate, and the remaining 28 older adults (9 males and 19 females; mean age = 69.43 years; SD = 6.77) participated in this study. The majority of the older adults were married Chinese Singaporeans who had retired and were living with others (family members). Only 21 older adults (control group: *n* = 11; intervention group: *n* = 10) were included in the analysis as they formed the first batch of older adults who received the WIPT intervention; the second batch (*n* = 7; control group: *n* = 3; intervention group: *n* = 4) of older adults could not proceed with the study due to the COVID-19 pandemic and the cessation of the study. All the older adults from the first batch of the intervention group had at least a 70% attendance rate (attended at least five sessions) for the seven-session intervention, and their absences were mainly due to other compelling commitments (sudden change in medical appointments or family matters). At the 3- and 6-month follow-up sessions, one and five older adults dropped out at each follow-up session, respectively, due to work commitments. This resulted in the analysis of 20 (95.2%) and 16 (76.2%) older adults at the respective time points for the final questionnaire analysis. In terms of blood sample analysis, blood samples of the 15 (71.4%) older adults from the first batch were analyzed; blood samples of participants who did not undergo the 6-month follow-up were excluded. The study participant flowchart and characteristics are shown in Figure 1 and Table 1, respectively. 

### 3.2. Preliminary Efficacy

This study investigated the effect of the trained lay volunteer-led community-based intervention on older adults’ levels of depression (GDS), anxiety (GAI), life satisfaction (SWLS), social connectedness (FS), and quality of life (WHOQOL-OLD BREF). The data were counted and rank-ordered for Mann–Whitney *U* tests. There were no baseline sociodemographic differences between the control and intervention groups as seen in Table 1. At baseline, the results also showed no significant differences between the control and intervention groups in terms of older adults’ levels of depression, anxiety, life satisfaction, and quality of life (all *p* > 0.05). However, the results showed that older adults in the intervention group (median = 22.50) scored significantly lower on the FS than the control group (median = 27.00), *U* = 24.00, *p* < 0.05 (Table 2). Thus, at the basal analysis, the intervention had a lower level of social connectedness than the control group. 

Based on the between-group analyses, there were no significant differences in change in outcome scores from baseline to 3 months for depression, anxiety, life satisfaction, friendship satisfaction, and quality of life between the intervention and control groups. Very small effect sizes were found for all measured outcomes. No significant difference in change in outcome scores from baseline to 6 months was found between the control and intervention groups for all outcomes as well. The very small effect size suggests that the changes in outcomes scores were minimally attributed to the intervention. The results are presented in Table 3.

Based on the Wilcoxon signed-rank test, no significant differences were found for change in scores for all outcomes from baseline to 3 months for both intervention and control groups. However, from baseline to 6 months, there is a significant change in outcome score for quality of life in older adults in the intervention group. No other within-group differences were observed for depression, anxiety, life satisfaction, and friendship (all *p* > 0.05). The summary data of the within-group differences of change in outcome scores from baseline to 3 months and baseline to 6 months are shown in Table 4 and Table 5, respectively. 

### 3.3. Stress- and Anxiety-Related Inflammatory Outcomes

Three markers from the blood samples were examined—cortisol, annexin-A1, and interleukin-1β. In the control group, eight out of nine older adults had increased cortisol levels, resulting in a significant increase in cortisol level from baseline (82 ± 14 ng/mL) to the 6-month follow-up (107 ± 13 ng/mL; *p* < 0.05). Thus, the control group showed a significant increase in stress levels from baseline to the 6-month follow-up. All nine older adults showed decreased annexin-A1 levels resulting in a significant decrease in annexin-A1 level from baseline (32 ± 4 ng/mL) to the 6-month follow-up (12 ± 2 ng/mL; *p* < 0.05). Thus, the control group showed a significant decrease in anxiety levels from baseline to the 6-month follow-up. The control group did not show a significant difference in the interleukin-1β levels between the baseline and 6-month follow-up. In the intervention group, there were no significant differences in the older adults’ cortisol, annexin-A1, and interleukin-1β levels between the baseline and 6-month follow-up. Table 6 shows the summary data of the blood sample outcomes. 

### 3.4. Qualitative Findings

A total of 6 older adults (1 male, 5 females) from the 10 adults who received the intervention, with a mean age of 74.60 years (SD = 6.28), participated in the individual face-to-face interview. The six interviews gathered a range of experiences from those who attended the WIPT intervention. The thematic analysis revealed three themes: (a) impact on older adults’ well-being, (b) attitudes toward intervention, and (c) a way forward. 

#### 3.4.1. Impact on Older Adults’ Well-Being

The older adults expressed an improvement in their overall well-being. They experienced improved moods and decreased negative feelings, in which they feel “more in control”, more “confident”, and “more present” and “become more aware” of the things happening around them. They felt that they had become “less fussy” and “more open-minded” and had developed a more positive outlook in life. Some older adults expressed that the intervention “reinforced” their habits and allowed them to pick up meditation again. The intervention enabled them to forge bonds and form a larger social circle. They began to “open up” to their friends and “socialize more”, were able to “empathize” with others better, and felt that they had “become a better person”. 

#### 3.4.2. Attitudes toward Intervention

All the older adults generally displayed a positive attitude towards the intervention as they felt “satisfied” and “pleased” with the experience. They found the intervention sessions “easy to follow and understand”, and they “look forward to coming to these sessions”. They described the facilitators as “engaging” and “professional”, and they were pleased with the handouts provided. All of them felt that it was “worth their time” to participate in the intervention. However, they felt that the “intervention was too short”, and the project and content for the sessions “lacked depth in information”. 

#### 3.4.3. A Way Forward

The older adults provided some suggestions to improve the intervention. One older adult proposed using advertising materials, such as brochures, to reinforce the objectives and intention of the project and appeal to interested participants. Some felt that participants should be grouped based on their backgrounds (education qualification) for standardization. They would also prefer more sessions with more in-depth information about mindfulness and mental well-being as they “would like to proceed and advance further”. One older adult suggested a recap of the mindfulness exercise learned at the end of each session for “reinforcement”. Two older adults suggested larger fonts on the handouts for readability and preferred for all the handouts to be distributed to them at once rather than separately at each session. One older adult also suggested the integration of “different religious ways of awareness” to learn more about the different ethnic groups in Singapore. 

## 4. Discussion

This study examined the preliminary efficacy of the intervention provided by lay trainers to older adults aged 60–95 years with subsyndromal depression and/or anxiety. The primary outcomes were depression and anxiety, and the secondary outcomes included life satisfaction, social connectedness (friendship), and quality of life. Stress- and anxiety-related inflammatory outcomes (cortisol, annexin-A1, and interleukin-1β) were measured, and the older adults’ perceptions and acceptability of the intervention were gathered through semistructured interviews. 

The lack of between-group significance in score change and the very small effect sizes for all outcomes suggest that the change in scores for depression, anxiety, life satisfaction friendship, and quality of life were minimally attributed to the intervention. However, within the intervention group, there was a significant positive change in scores for quality of life from baseline to 6 months, which suggests that despite the absence of immediate effects, there may be long-term benefit of the intervention in enhancing perceived quality of life among older adults. This finding corresponds with a previous study that found within-group improvement of quality of life among older adults who received a mindfulness-based intervention [40]. Another review also reported positive benefits of SFBT on aging and quality of life [21]. However, as this is a unique hybrid intervention, more research is warranted to confirm the findings. 

In this study, the stress-related biomarkers used were cortisol, annexin-A1, and interleukin-1. Our study demonstrated that the control group had significantly higher cortisol levels and lower annexin-A1 levels at 6 months, while the intervention group did not. It is known that stress induces cortisol, an inflammation regulator, through the hypothalamic–pituitary–adrenal axis [41]. Chronically high levels of cortisol may lead to unresolved inflammation, which can be monitored with annexin-A1, resulting in conditions such as diabetes, stroke, and obesity [13,14]. Therefore, it is plausible that the WIT intervention might be effective in reducing the risk of increased stress among older adults.

The lack of between-group differences observed in the majority of the outcomes could be due to various factors: (1) recruitment and small sample size, (2) limited power of nonparametric tests, (3) attrition rates, and (4) intervention characteristics. Alike other research studies, recruiting participants was a challenge. Many older adults cited work commitments and disinterest in the study as reasons for declining to participate. To improve recruitment, one staff member could be designated to focus on recruitment only [32]. The staff would need to address mental health issues delicately and highlight the benefits of participating in the intervention.

Small sample sizes decrease the study’s statistical power and increase the risk of type II error [42]. Although the nonparametric tests were appropriate, they observe fewer assumptions and are generally less powerful than parametric tests, hence increasing the chance of type II error [43]. Moreover, despite a minimum of 50% attendance for all seven sessions, attrition rates at 3- and 6-month follow-up sessions were 5% and 25%, respectively. As a rule of thumb, <5% attrition leads to minimal bias, while >20% attrition could pose serious attrition bias (i.e., systematic differences between people who leave the study and those who continue) and compromise the validity of the study’s results [44]. However, the seemingly high attrition rate in this study may be attributed to the small sample size, which is a significant study limitation. Considering that the small sample size, nature of nonparametric tests, and high attrition rates could have skewed the current results, these results should be interpreted with caution. Furthermore, older adults cited the common reason of scheduling difficulties to explain their absences [45]. Hence, it is imperative to reinforce and clarify the expected commitments to the study intervention (e.g., number of visits) before recruiting the participants so that any potential challenges in committing to the study could be addressed in time. To maximize attendance rates, more intervention sessions could be scheduled or alternative delivery platforms (e.g., Zoom webinars) could be adopted to offer participants more convenient timeslots. As web-based educational programs and mindfulness training have proven effective and acceptable among older adults [46,47,48], future studies should consider delivering interventions online to increase accessibility and maximize the cost-effectiveness of the study. Furthermore, intervention characteristics such as intervention delivery method, intervention length and frequency, deliverer–participant fit, and the environment where the intervention is conducted could affect the overall efficacy and validity of the intervention [49].

Consistent with the quantitative findings on improved perceived quality of life at 6 months among older adults in the intervention group, older adults who participated in the intervention generally reported positive effects on their well-being (e.g., improved moods, became more open-minded and sociable) during the semistructured interviews. They found the intervention worthy of their time and looked forward to the sessions. Their responses were similar to another lay-led community-based mental health intervention with qualitative findings that reported reduced depressive moods and improved social functioning in adults [50]. They also suggested changing the recruitment process to appeal to more potential participants and have more informative and mindfulness training sessions for better insight. Overall, older adults viewed the intervention positively and were keen to participate in it.

### Strengths, Limitations, and Future Implications

This study included the detailed tracking and holistic assessments (mixed methods and blood assessments) of a sample of community-dwelling older adults. The findings highlighted the plausibility and potential for the expansion of the one-of-a-kind WIPT intervention, an intervention that combines SFBT and mindfulness, to support a portion of the population who are at risk of mental health conditions and require outreach and nonprofessional support. The pilot data stemming from a smaller sample size will also provide effect estimates for future RCT studies that intend to evaluate this intervention. Moreover, bias could be eliminated by using the gold standard for effectiveness research—RCT research design. However, the study’s small sample size, high attrition rates, and use of nonparametric tests prevented the making of definite and firm conclusions about the effectiveness of the intervention based on the preliminary efficacy assessments. All these factors, coupled with the predominantly female sample recruited in this study, could limit the generalizability of this study’s findings. Additionally, the use of the GDS score range of 1 to 5 to detect SSD may result in likely floor effects as there is not much noticeable difference in someone who has a score of 1 on the GDS. Another limitation is using a single blood draw to measure biomarkers, especially cortisol, which has a diurnal pattern. Future studies involving biomarkers should consider having one blood draw in the morning and another in the afternoon to examine the change in levels. Conversely, the qualitative interviews highlighted the intervention’s acceptability and provided suggestions for improvement. Future research could improve the recruitment efforts and reinforce the importance of committing to the intervention sessions to increase attendance rates. Alternative delivery platforms such as the online medium could be considered in the future to increase the accessibility and flexibility of the intervention sessions.

## 5. Conclusions

Community-dwelling older adults often go unnoticed, and outreach is limited in the local context. It is essential to provide timely support to prevent the deterioration of their overall well-being. In addition to the promising results on quality-of-life enhancement and reduced risk of elevated stress, this pilot study documented areas for improvement and refinement for future similar interventions. Overall, the qualitative findings highlighted general favorable and positive experiences of the older adults and identified suggestions for improvement. Moreover, future studies need to consider the long-lasting adverse psychological effects that the COVID-19 pandemic could have on adults’ mental health and overall well-being. Hence, additional efforts should be made during and after the pandemic to reach out to community-dwelling older adults, especially those who live alone as they may be more susceptible to developing mental health conditions.

## Figures and Tables

**Table 1:** Baseline characteristics of participants.

Characteristics	Total (*n* = 28), *n*(%)	Intervention (*n* = 14), *n*(%)	Control (*n* = 14), *n*(%)	*p*-Value
Age (mean (SD), range)	69.4 (6.8), 61—83	71.1 (5.9), 62—82	67.7 (7.4), 61—83	0.185
Gender				
Male	9 (32.1)	5 (35.7)	4 (28.6)	0.686
Female	19 (67.9)	9 (64.3)	10 (71.4)	
Ethnicity				
Chinese	26 (92.9)	14 (100)	12 (85.7)	0.341
Indian	1 (3.6)	0 (0)	1 (7.1)	
Others	1 (3.6)	0 (0)	1 (7.1)	
Marital status				
Single	1 (3.6)	1 (7.1)	0 (0)	0.572
Married	22 (78.6)	11 (78.6)	11 (78.6)	
Widowed	4 (14.3)	2 (14.3)	2 (14.3)	
Others	1 (3.6)	0 (0)	1 (7.1)	
Education level				
None	1 (3.6)	1 (7.1)	0 (0)	0.504
Primary	3 (10.7)	1 (7.1)	2 (14.3)	
Secondary	9 (32.1)	3 (21.4)	6 (42.9)	
ITE/Poly/JC	6 (21.4)	3 (21.4)	3 (21.4)	
University	9 (32.1)	6 (42.9)	3 (21.4)	
Current employment status				
Self-employed	2 (7.1)	1 (7.1)	1 (7.1)	0.699
Full-time	1 (3.6)	0 (0)	1 (7.1)	
Part-time	1 (3.6)	0 (0)	1 (7.1)	
Unemployed	4 (14.3)	2 (14.3)	2 (14.3)	
Retired	20 (71.4)	11 (78.6)	9 (64.3)	
Living arrangement				
Alone	1 (3.6)	1 (7.1)	0 (0)	0.494
With spouse	15 (53.6)	7 (50.0)	8 (57.1)	
With children only	6 (21.4)	4 (28.6)	2 (14.3)	
With others	6 (21.4)	2 (14.3)	4 (28.6)	
Mobility status				
Independent	25 (89.3)	12 (85.7)	13 (92.9)	0.541
Independent with walking aid	3 (10.7)	2 (14.3)	1 (7.1)	
Medical comorbidities				
None	13 (46.5)	4 (28.6)	9 (64.3)	0.062
1–2	12 (42.9)	9 (64.3)	3 (21.4)	
≥3	3 (10.7)	1 (7.1)	2 (14.3)	

Note. ITE: Institute of Technical Education; JC: junior college; SD: standard deviation. ANOVA was performed for continuous variables and chi-square was performed for categorical variables.

**Table 2:** Between-group differences in outcome scores at baseline.

Scale	Medians (IQR: Q1, Q3)		*U*	*p*
	Control Group (*n* = 11)	Intervention Group (*n* = 10)		
GDS	1.00 (0.00, 3.00)	1.50 (0.75, 5.00)	44.50	0.47
GAI	0.00 (0.00, 3.00)	2.50 (0.75, 9.00)	32.00	0.11
SWLS	28.00 (20.00, 28.00)	24.50 (18.75, 27.00)	39.50	0.28
FS	27.00 (25.00, 30.00)	22.50 (18.75, 25.50)	24.00 *	0.03
WHOQOL-OLD	51.00 (46.00, 52.00)	49.50 (43.75, 51.75)	44.01	0.47

Note. * *p* < 0.05; IQR: interquartile range; Q1: 25th percentile; Q3: 75th percentile; GDS: Geriatric Depression Scale; GAI: Geriatric Anxiety Inventory; SWLS: Satisfaction with Life Scale; FS: Friendship Scale; WHOQOL-OLD: World Health Organization Quality of Life OLD.

**Table 3:** Between-group differences for change in outcome scores from baseline to 3 months and baseline to 6 months.

Scale	Medians (IQR: Q1, Q3)		U	*p*	Effect Size [95% CI]
Baseline to 3 months	Control (*n* = 11)	Intervention (*n* = 9)			
GDS	0.00 (0.00, 2.00)	–1.00 (–2.50, 1.50)	34.0	0.231	0.072 [–2, 4]
GAI	0.00 (0.00, 0.00)	0.00 (–3.00, 0.00)	36.0	0.261	0.063 [0, 3]
SWLS	0.00 (–2.00, 0.00)	–1.00 (–2.50, 2.00)	47.5	0.878	0.001 [–3, 3]
FS	–1.00 (–2.00, 0.00)	–1.00 (–2.50, 2.00)	48.0	0.908	0.001 [–4, 2]
WHOQOL-OLD	0.00 (–4.00, 0.00)	1.00 (–3.00, 3.00)	35.0	0.266	0.062 [–7, 3]
**Baseline to 6 months**	**Control (*n* = 10)**	**Intervention (*n* = 6)**			
GDS	0.00 (–1.00, 2.00)	0.00 (–1.25, 2.00)	29.0	0.912	0.001 [–5, 3]
GAI	0.00 (–1.25, 1.25)	–0.50 (–1.25, 1.25)	27.5	0.782	0.005 [–2, 2]
SWLS	–1.00 (–2.00, 2.50)	0.50 (–2.50, 6.75)	26.0	0.661	0.012 [–7, 3]
FS	0.00 (−3.25, 1.25)	0.50 (–1.25, 1.50)	25.5	0.622	0.015 [–4, 2]
WHOQOL-OLD	–1.50 (–4.25, 1.50)	1.00 (0.75, 2.50)	16.0	0.123	0.149 [–6, 1]

Note. IQR: interquartile range; Q1: 25th percentile; Q3: 75th percentile; 95% CI: 95% confidence interval; GDS: Geriatric Depression Scale; GAI: Geriatric Anxiety Inventory; SWLS: Satisfaction with Life Scale; FS: Friendship Scale; WHOQOL-OLD: World Health Organization Quality of Life OLD.

**Table 4:** Within-group difference for change in outcome scores from baseline to 3 months.

Scale	Negative			Positive			Test Statistic		
	*n*	Mean Rank	Sum of Ranks	*n*	Mean Rank	Sum of Ranks	Ties	Z	*p*
**Control (*n* = 11)**									
GDS	2	4.50	9.0	5	3.80	19.0	4	–0.85	0.40
GAI	2	3.00	6.0	2	2.00	4.0	7	–0.37	0.72
SWLS	5	3.90	19.5	2	4.25	8.5	4	–0.94	0.35
FS	6	4.25	25.5	2	5.25	10.5	3	–1.07	0.29
WHOQOL	5	7.00	35.0	5	4.00	20.0	1	–0.77	0.44
**Intervention (*n* = 9)**									
GDS	5	3.40	17.0	2	5.50	11.0	2	–0.51	0.61
GAI	4	42.75	11.0	1	4.00	4.0	4	–0.95	0.34
SWLS	5	4.10	20.5	3	5.17	15.5	1	–0.35	0.72
FS	5	3.10	15.5	2	6.25	12.5	2	–0.25	0.80
WHOQOL-OLD	3	5.17	15.5	5	4.10	20.5	1	–0.35	0.73

Note. FS: Friendship Scale; GAI: Geriatric Anxiety Inventory; GDS: Geriatric Depression Scale; SWLS: Satisfaction with Life Scale; WHOQOL: World Health Organization Quality of Life OLD.

**Table 5:** Within-group difference for change in outcome scores from baseline to 6 months.

Scale	Negative			Positive			Test Statistic		
	*n*	Mean Rank	Sum of Ranks	*n*	Mean Rank	Sum of Ranks	Ties	Z	*p*
**Control (*n* = 10)**									
GDS	3	3.00	9.0	3	4.00	12.0	4	–0.32	0.75
GAI	3	3.67	11.0	3	3.33	10.0	4	–0.11	0.92
SWLS	6	4.33	26.0	3	6.33	19.0	1	–0.42	0.68
FS	4	5.00	20.0	4	4.00	16.0	2	–0.28	0.78
WHOQOL	6	5.83	35.0	4	5.00	20.0	0	–0.77	0.44
**Intervention (*n* = 6)**									
GDS	3	3.33	10.0	3	3.67	11.0	0	–0.11	0.91
GAI	3	2.83	8.5	2	3.25	6.5	1	–0.28	0.78
SWLS	3	2.50	7.5	3	4.50	13.5	0	–0.63	0.53
FS	2	3.00	6.0	3	3.00	9.0	1	–0.41	0.68
WHOQOL-OLD	0	0.00	0.00	5	3.00	15.0	1	–2.06	0.04 *

Note. * *p* < 0.05; FS: Friendship Scale; GAI: Geriatric Anxiety Inventory; GDS: Geriatric Depression Scale; SWLS: Satisfaction with Life Scale; WHOQOL: World Health Organization Quality of Life OLD.

**Table 6:** Summary data of blood sample analyses at baseline and 6-month follow-up.

Markers		Control Group			Intervention Group			
	Baseline	6-Month Follow-Up	Difference	*p*	Baseline	6-Month Follow-Up	Difference	*p*
Cortisol (ng/mL)	82 ± 14	107 ± 13	+25	0.02	74 ± 16	92 ± 12	+18	0.30
Annexin-A1 (ng/mL)	32 ± 4	12 ± 2	−20	0.002	28 ± 6	14 ± 4	−14	0.16
Interleukin-1β (pg/mL)	4 ± 1.5	3 ± 0.7	−1	0.45	6.6 ± 2.3	2.4 ± 0.7	−4.2	0.14

## Data Availability

The data presented in this study are available in this article and its supplementary material.

## Supplementary Materials

The following are available online at https://www.mdpi.com/article/10.3390/ijerph18189514/s1, Table S1: Outline of the WIPT intervention.
