# Supplementary material for: “Where-There-Is-No-Psychiatrist Integrated Personal Therapy” among Community-Dwelling Older Adults: A Randomized Pilot Study
Source: Int J Environ Res Public Health. 2021 Sep 9;18(18):9514. doi: 10.3390/ijerph18189514 (PMC8468930; doi:10.3390/ijerph18189514)
Supplement: Supplementary file 1 [file ijerph-18-09514-s001.zip › ijerph-1261805-supplementary.pdf]

Table S1. Overview and timeline of the WIPT Intervention.

Overview of the WIPT intervention	
**Research team**	Consist of content experts in psychoeducation, psychology, psychiatry, and mindfulness training
**Topics overview**	Depression- and anxiety-related information, types of coping strategies, importance of social support from the community, and awareness to acknowledge a problem and seek help
**Intervention frequency and duration**	<u>3 months (7 sessions):</u><br>- First month (4 weekly sessions)<br>- Second month (2 bi-weekly sessions)<br>- Third month (1 session on the last week of the month)<br><br><u>1 session (90minutes):</u><br>- 30 minutes of mindfulness training<br>- 60 minutes of solution focused brief therapy (SFBT)<br>• Psychoeducation<br>• Structured life review therapy - involves a structured evaluation of one's past experiences and future goal setting
**Timeline**	**Activity**
Week 1	Mindfulness Activity (30mins):<br>• Introduction to mindfulness<br>• Mindful eating<br>PowerPoint Slides (60mins):<br>1) Introduction to WIT<br>• What is expected in the coming weeks<br>• Participants' introductions and ice-breaking exercise<br>• Ground rules<br>2) Education on Depression and Anxiety<br>• Introduction, prevalence, symptoms, and coping of the conditions
Week 2	Mindfulness Activity (30mins):<br>• Mindful breathing<br>PowerPoint Slides (60mins):<br>1) Introduction to Solution-Focused Brief Therapy (SFBT)<br>• Recap of Week 1 and key messages by participants<br>• SFBT (search inside yourself)<br>• Enquire about the time participants felt emotionally down

- Any solutions they looked for and how did they cope?
- Explore the time participants felt more or less emotionally down; any different peculiarities

2) Education on help-seeking behaviours and social integration
    - Seek help from friends and family
    - Discuss the available resources
    - Discuss on caring about others, vice versa
    - Feelings of belonging to a group or community

Week 3 Mindfulness Activity (30mins):

- Mindful movement or walking

PowerPoint Slides (60mins):

1) Introduction to active ageing
    - Recap of Week 2 and key messages by participants
    - Characteristics of active ageing:
        - Exercise
        - Inter-generational connectedness
        - Holistic well-being (mind and body)
2) Social connectedness
    - Discuss relationships over the life course (i.e., people they met and the ones they cherish, why so, how did they make them feel?)
    - Discuss quality/quality of relationships
    - Get participants to bring along objects of sentimental value for next session

Week 4 Mindfulness Activity (30mins):

- Body scan

PowerPoint Slides (60mins):

1) Introduction to reminisce
    - Recap of Week 3 and key messages by participants
    - Discuss on objects brought by the participants
    - Write down personal thoughts/share about the objects and how they enhance sense of belonging
    - How can these objects be used to make you feel better?
2) Community and your role
    - Discuss on living environments
    - Sense of belonging to the community

- How it differs from the past and how can we cultivate the "kampong spirit"?
- Elements of the "kampong spirit"?
- How can you get the community to help with your low moods/emotions?

3) Homework:
   - Take a log on what are the small steps you can take to alleviate low moods?
   - Stay socially active with the peers from this group

Week 5 No face-to-face session

Week 6 Mindfulness Activity (30mins):

- Allowing and letting go (exploring difficulty)

PowerPoint Slides (60mins):

1) Case study discussion
   - Actual case of an older adult who went through depression
   - Discuss the following:
     - How did the case cope with depression?
     - Sources of strength and motivation?
     - How did the case overcome his/her weakness?
2) Reinforcement on Week 1
   - Understanding depression
   - Introduction, prevalence, symptoms, and coping of the condition

Week 7 No face-to-face session

Week 8 Mindfulness Activity (30mins):

- Loving kindness

PowerPoint Slides (60mins):

1) Personal sharing of hobbies
2) Strategies to cope with low moods/depression/anxiety
   - Sharing of strategies by facilitator
   - Sharing of strategies by participants
   - Group discussion on what would work for the participants

Week 9 No face-to-face session

Week 10 No face-to-face session

Week 11 No face-to-face session

Week 12 Mindfulness Activity (30mins):

- Allow participants to choose the activity they want to practise

- Gratitude practice

PowerPoint Slides (60mins):

1) Learning points from intervention
    - Sharing by participants
    - Feedback/suggestions
2) Group activities (Games)
    - Memory games
    - Jenga
